# Stability analysis on dark solitons in quasi-1D Bose–Einstein condensate with three-body interactions

**DOI:** 10.1038/s41598-021-90814-2

**Published:** 2021-05-31

**Authors:** Yushan Zhou, Hongjuan Meng, Juan Zhang, Xiaolin Li, Xueping Ren, Xiaohuan Wan, Zhikun Zhou, Jing Wang, Xiaobei Fan, Yuren Shi

**Affiliations:** 1 College of Physics and Electronic Engineering, Northwest Normal University, Lanzhou, 730070 People’s Republic of China; 2 Laboratory of Atomic Molecular Physics and Functional Material, Northwest Normal University, Lanzhou, 730070 People’s Republic of China

**Keywords:** Ultracold gases, Computational science

## Abstract

The stability properties of dark solitons in quasi-one-dimensional Bose–Einstein condensate (BEC) loaded in a Jacobian elliptic sine potential with three-body interactions are investigated theoretically. The solitons are obtained by the Newton-Conjugate Gradient method. A stationary cubic-quintic nonlinear Schrödinger equation is derived to describe the profiles of solitons via the multi-scale technique. It is found that the three-body interaction has distinct effect on the stability properties of solitons. Especially, such a nonlinear system supports the so-called dark solitons (kink or bubble), which can be excited not only in the gap, but also in the band. The bubbles are always linearly and dynamically unstable, and they cannot be excited if the three-body interaction is absent. Both stable and unstable kinks, depending on the physical parameters, can be excited in the BEC system.

## Introduction

Bose–Einstein condensate (BEC) is an interesting physical phenomenon, which was originally predicted by Bose and Einstein in 1924. Since the first successful experimental realization of BECs^1–3^, a large number of theoretical and experimental^4–7^ interests in this field has been attracted in recent years such as atomic lasers, vortices, vortex array, quantum phase transition, and so on. It is well known that the dynamics of BECs are usually described by the nonlinear Gross-Pitaevskii equation (GPE) under mean-field approximation at extremely low temperature^8–10^. At low density, where interatomic distances are much greater than the distance scale of atom-atom interactions, we can use a single parameter (scattering length) to describe the two-body interaction^11^. However, when the two-body interaction increases, the central density of the condensate will be higher, then the three-body interaction should be taken into account. Consequently, the three-body interaction become meaningful and account for the quintic mean-field nonlinearity. The existence of three-body interactions also play an important role in the condensate’s stability^12–15^. The dynamics can be described by a set of nonlinear Schrödinger equation (NLSE) , which is integrable^16^, with two- and three-body interactions^4^. Applications of cubic-quintic NLSE are not limited to condensate mater problems. Recently, both experimentally and theoretically, the three-body interaction could be observed or realized^17–19^. In particular, the investigations of the linear stability properties of BECs with two- and three-body interactions have been a significant interest in this topic^11^.

The discovery of solitons plays a milestone role in the development of nonlinear physics. In BECs, both experiments and theoretical studies have found bright solitons^20^, dark solitons^21^, vortex solitons^22^, vortex lattice^23^ and so on. Moreover, periodic media in BEC generated by optical methods, such as crystal lattice, is the most familiar classical example^24^. Ref.^25^ investigated the possibility of solitary bound formation between spin clusters and lattice deformation. Whether in BEC or optics, the GPE can be used to describe the nonlinear dynamical behaviors. It is also known that many periodic potentials are adopted as trigonometric functions^26–31^. Especially, there are all kinds of localized nonlinear modes of condensates that cannot exist in the linear limit. Here, they are located in the band-gaps of the matter-wave spectrum, therefore they can be called gap solitons^32^. They can also exist in different types of nonlinear periodic structures including optics^33,34^, BEC^35,36^, and so on. The cubic-quintic NLSE possesses both bright and dark solitons. The bright one is known already from the work of Pushkarov et al.^37^. The dark solitons are another kind of topological solitons with the non-vanishing boundary conditions. They can be clarified into two kinds: kink soliton and gray soliton. In literatures, the gray soliton is usually named as the bubble-like soliton. The repulsive cubic NLSE does not have solutions of this kind^38^. Due to the fifth-order nonlinearity in a fibre, bright and dark solitons can propagate in the same parameter range^39^. In Ref.^40^, the authors investigated the dynamical stability properties of the cubic NLSE with a Jacobian elliptic potential in a quasi-one-dimensional (1D) BEC. They have presented an analysis that contains analytical existence criteria for solitons of the cubic-quintic NLSE and exact analytical expressions for the intensity, phase, and normalized momentum in Ref.^41^. However, by now, there are less works on investigating the linear stability properties of dark solitons in a quasi-1D BEC loaded in a Jacobian elliptic potential with three-body interactions. In this paper, we will mainly pay attention to such an interesting work.

The remaining contents are arranged as follow. In “Model” section, we made an introduction to the theoretical model for a quasi-1D BEC with two- and three-body interactions. In “Solitons and their stability properties” section, we firstly numerically found various solitons by the Newton-Conjugate Gradient (NCG) method. Secondly, the multi-scale technique is applied to theoretically analyse the solitons. Finally, we numerically study the stability properties of the solitons. In “Conclusion” section, some conclusions are summarized.

## Model

At ultra-low temperatures, the dynamical behaviors of BECs with two- and three-body interactions can be described by the following three-dimensional (3D) nonlinear GPE^11^ with two-body and three-body interaction

1 $$\begin{aligned} i\hbar \frac{\partial \Psi }{\partial t}=-\frac{\hbar ^2}{2m}{\nabla ^2}\Psi +V(\mathbf {r})\Psi +g_1|\Psi |^2\Psi +g_2|\Psi |^4\Psi , \end{aligned}$$

where $\Psi =\Psi (\mathbf {r},t)$ labels the condensate wave function, $V(\mathbf {r})$ is an experimentally generated macroscopic potential, $\mathbf {r}=(x,y,z)$ the Cartesian coordinate vector, $\nabla ^2$ the Laplacian operator, $\hbar$ the reduced Planck constant, *m* the mass of the atom. $g_1=\frac{4\pi \hbar ^2a_s}{m}$ denotes the strength of two-body interaction. $a_s$ is the s-wave scattering length ($a_s>0$ and $a_s<0$ respectively represents the repulsive and attractive interaction), which can be tuned to any desired value by using the “Feshbach resonance” technique^42^. $g_2$ is the strength of the three-body interaction. In Ref.^43^, $g_2$ is given by a universal formula $g_2=\frac{12\pi \hbar ^2a_s^2}{m}\left[ d_1+d_2{\mathrm {tan}}\left( s_0\ln \frac{|a_s|}{|a_0|}+\frac{\pi }{2}\right) \right]$, where the numerical values of the universal constants $d_1,d_2,a_0$ and $s_0$ are given in Refs.^43,44^. From Ref.^43^, we know that $g_2$ can be tuned from $-\infty$ to $+\infty$. The total atom particles are $N=\int |\Psi |^2d^3\mathbf {r}$.

In experiments, the BEC atoms are usually confined in a harmonic potential $V(\mathbf {r})=\frac{1}{2}m(\omega _x^2x^2+\omega _y^2y^2+\omega _z^2z^2)$ with $\omega _x$, $\omega _y$ and $\omega _z$ being the trap frequencies along *x*, *y* and *z*-directions. In the disk-shaped condensates, i.e., $\omega _x\approx \omega _y$ and $\omega _z\gg \omega _x$, the 3D GPE can be reduced to 2D GPE. In the cigar-shaped condensates, i.e., $\omega _y,\omega _z\gg \omega _x$, the 3D GPE can be reduced to 1D GPE. In this paper, we only consider the cigar-shaped condensate. By introducing the dimensionless variables ${\tilde{t}}=\omega _xt$, ${\tilde{x}}=x/a_{\mathrm {h0}}$, $\tilde{\Psi }=\sqrt{a_{\mathrm {h0}}^3/n_0}\Psi$, $\tilde{g}_1=\frac{2a_sn_0\sqrt{\omega _z\omega _y}}{a_{\mathrm {h0}}\omega _x}$, $\tilde{g}_2=\frac{n_0\omega _z\omega _y}{3\pi ^2a_{\mathrm {h0}}^3\hbar \omega _x^3}{g_2}$, where $a_{\mathrm {h0}}=\sqrt{\hbar /{m\omega _x}}$ is the characterized length of harmonic oscillator, $n_0$ is a given particle density. In general, one can take $n_0=N$ (This is not necessary.). By using the approach presented in Ref.^45^ and omitting the tilde $'\sim '$ above all the variables, one can obtain the following quasi-1D dimensionless GPE

2 $$\begin{aligned} i\frac{\partial \Psi }{\partial t}=-\frac{1}{2}\frac{\partial ^2\Psi }{\partial x^2}+V(x)\Psi +g_1|\Psi |^2\Psi +g_2|\Psi |^4\Psi . \end{aligned}$$

Under which, the total particle number can be expressed as $N=n_0\int {|\Psi (x,t)|^2dx}$. We choose the periodic external potential as $V(x)=V_0{\mathrm {sn}}^2(x,q)$, where ${\mathrm {sn}}(x,q)$ is the Jacobian elliptic sine function with modulus $q (0\le q <1)$. This potential can be regarded as a generalization to the trigonometry function. In experiments, such a potential can be well approximated by using only two laser beams^46,47^.

Solitary waves of Eq. (2) are sought in the form

3 $$\begin{aligned} \Psi (x,t)=\psi (x)e^{-i\mu t}, \end{aligned}$$

where $\mu$ is the chemical potential, $\psi (x)$ is a real-valued function, which satisfies the equation

4 $$\begin{aligned} \frac{1}{2}{\psi _{xx}}-V(x)\psi +\mu \psi -g_1\psi ^3-g_2\psi ^5=0. \end{aligned}$$

Note that if $\psi =\varphi (x)$ is an exact solution of Eq. (4), then so does $\psi =-\varphi (x)$. When $\psi (x)$ is infinitesimal, the terms $\psi ^3$ and $\psi ^5$ in Eq. (4) can be neglected, which results in a linear equation

5 $$\begin{aligned} \frac{1}{2}{\psi _{xx}}-V(x)\psi +\mu \psi =0. \end{aligned}$$

The bounded solutions of Eq. (5) are called linear Bloch modes, and the corresponding constant $\mu$ forms linear Bloch bands. Since *V*(*x*) is a periodic function, Eq. (5) is a generalized form of Mathieu’s equation. Its bounded solution can be written as^48^

6 $$\begin{aligned} \psi =p(x)={e^{ikx}}{\tilde{p}}(x;\mu ), \end{aligned}$$

where ${\tilde{p}}(x;\mu )$ has the same period as the potential *V*(*x*), $\mu =\mu (k)$ is the 1D dispersion relation. Both the linear Bloch bands and the dispersion relations can be gotten by solving the obtained eigenvalue problem. However, unfortunately, it is rather difficult to solve the eigenvalue problem exactly and analytically. Here, we solve it numerically by the Fourier collocation method^49^. The graph of Bloch band structures is omitted for it is similar as that shown in Ref.^46^.

## Solitons and their stability properties

When $\psi (x)$ is not infinitesimal, the terms $\psi ^3$ and $\psi ^5$ in Eq. (4) can not be neglected. Considering that the chemical potential $\mu$ enters into the band-gap, where the linear Bloch waves no longer exist, from the boundary of the band $k=k_0$, $\mu =\mu (k_0)$, completely localized solitary waves, namely gap solitons^32^, can be excited. The gap solitons can be found numerically by the NCG method^28^, which is an effective numerical method for seeking the solitary wave solutions of nonlinear evolution equations. It is based on Newton iteration and conjugate-gradient iteration method to solve the resulting linear equation. This method can be applied to compute both the ground states and excited states in various physical systems. More importantly, this method usually converges faster than the other numerical methods and it is easy to implement in Matlab. A detailed description about the NCG method can be found in Ref.^28^.

### Bright solitons

In literatures, the bright solitons are often defined as the solutions with vanishing boundary conditions, i.e. $\psi (\pm \infty )=0$. As we had done in Ref.^46^, we numerically find that virous of bright solitons (for examples, the on-site and off-site gap solitons) still exist when the three-body interaction is taken into account. Here we omitted the profiles of the bright solitons as they have the similar structures as those illustrated in Ref.^46^. We also noted that the amplitude of the bright solitons decreases when the three-body interaction strength $|g_2|$ increases.

**Figure 1:**
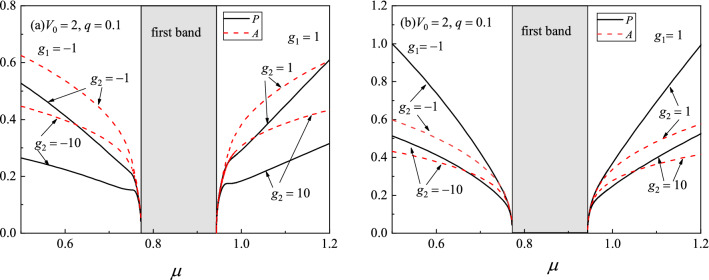
Amplitude (dashed lines) and power (solid lines) curves of gap solitons bifurcated from the first Bloch band. (**a**) on-site gap solitons. (**b**) off-site gap solitons.

To see it more clearly, we define the amplitude of bright soliton as $A=\max (|\psi |)$ and the particle number $P=\int {|\psi |^2dx}$, which implies that the total particles is $N=n_0P$. In nonlinear optics, *P* is often called the power. Figure 1 exhibits the amplitude and power of on-site and off-site solitons versus the chemical potential $\mu$ for different three-body interaction strength $g_2$. As can be seen from Fig. 1a and b, both *A* and *P* decrease when $\mu$ moves toward the first band for fixed $g_2$. In the semi-infinite gap, *P* linearly decreases when $\mu$ increases but far away from the band edge. However, *P* decreases rapidly if $\mu$ moves near the band. This is opposite in the first gap, i.e., *P* decreases linearly when $\mu$ decreases but far away from the first band edge. We also noticed from Fig. 1 that the larger the three-body interaction $|g_2|$, the smaller the amplitude *A* and the power *P*. This conclusion will be explained theoretically later.

**Figure 2:**
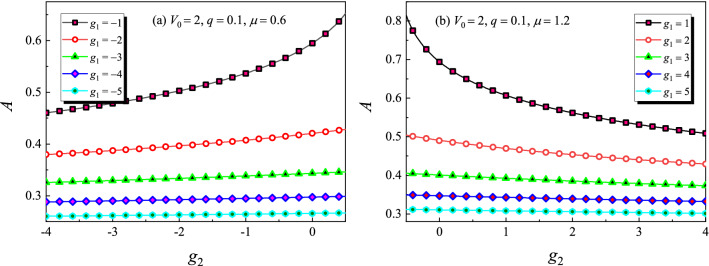
Amplitude of on-site gap solitons versus the three-body interaction strength $g_2$. (**a**) In the semi-infinite gap. (**b**) In the first gap.

Figure 2 shows the amplitude of on-site gap solitons versus the three-body interaction strength $g_2$ under different two-body interaction strength $g_1$. Other parameters are $V_0=2$ and $q=0.1$. For a fixed $g_2$, it is obvious that the larger the $|g_1|$ is, the smaller the amplitude of the solitons. In the semi-infinite gap, see Fig. 2a, the amplitude of solitons increases as $g_2$ increases for a given $g_1$. However, in the first gap, this is somewhat opposite. That is, see Fig. 2b, the amplitude of solitons decreases when $g_2$ increases. On the other hand, when the two-body interaction is stronger, i.e. $|g_1|$ is relatively larger, one can see from Fig. 2 that the amplitude of gap solitons nearly invariant with the increasing of $g_2$. This is because the amplitude of gap solitons is very small when $|g_1|$ is larger, so that the term $\psi ^5$ in Eq. (5) can be neglected. It is also noted that gap solitons indeed exist when $g_1g_2<0$ for a relatively narrow interval of $g_2$. We will make a theoretical explanation later.

### Dark solitons

Beyond the bright solitons discussed in the above section, the nonlinear Eq. (4) also has the so-called “dark solitons”. In literatures, the “dark soliton” is usually defined as the solutions with non-vanishing boundary conditions, i.e., $\psi (\pm \infty )\ne 0$. In Refs.^39^, a dark soliton is a “kink” when $\psi (-\infty )=-\psi (+\infty )$ and it is called a “gray soliton” when $\psi (-\infty )=\psi (+\infty )$. The gray soliton is also named as a “bubble-like soliton”. Here, for convenient, we call them as “kink” and “bubble”, respectively.

It is more difficult to find dark solitons than bright ones when the NCG method is applied. One must choose the initial guess carefully, or the iteration will be divergent or convergent to an unwanted result. In practice, we take the initial guess

$$\begin{aligned} \psi (x)=a_1\left( 1+0.04\cos \frac{2\pi x}{T_x}\right) \tanh x \end{aligned}$$

for seeking the kink and

$$\begin{aligned} \psi (x)=a_2\left( 1+0.04\cos \frac{2\pi x}{T_x}\right) (1-a_3e^{-x^2}) \end{aligned}$$

for the bubble, where $T_x$ is the periodicity of the external potential ($T_x\approx \pi$ when $q=0.1$). One can choose appropriate values for $a_1, a_2$ and $a_3$ to obtain the wanted results. Of course, the coefficient in front of the cosine function also can be adjusted if necessary.

**Figure 3:**
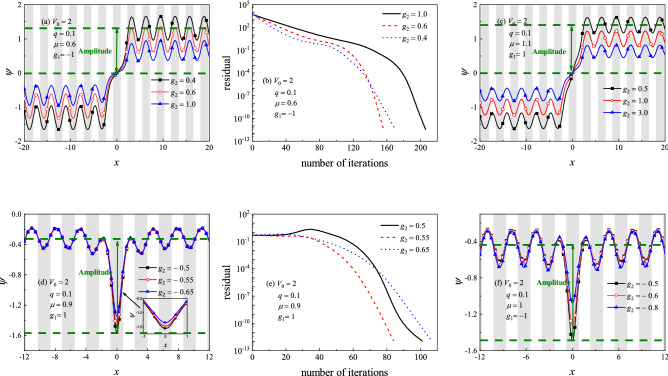
Profiles of dark solitons lie in (**a**) the semi-infinite gap, (**c**,**f**) the first gap, and (**d**) the first band, for $q=0.1$ with different nonlinear interaction strength. (**b**,**e**) Residual diagram of the NCG method versus the number of iterations for the dark solitons shown in (**a**) and (**d**), respectively. Shaded regions represent lattice sites, i.e., regions of low potential values *V*(*x*).

Figure 3 shows the profiles of kinks (Fig. 3a and c) and bubbles (Fig. 3d and f) given by the NCG method. The amplitudes of the dark solitons are defined as illustrated in Fig. 3, where the upper green dashed lines denotes the average value of the Bloch waves in one periodicity. One can see that the kink is odd symmetric, while the bubble is even symmetric. When |*x*| is large enough, the matter waves oscillate with the same periodicity as the external potential. This is because of the periodical carrier Bloch wave has been modulated by a kink or a bell-like soliton. Figure 3b and e show the residual error, measured as the maximum of the residue in Eq. (4), when the NCG method is applied to seek the dark solitons shown in Fig. 3a and d, respectively. It is seen that the residual drops below $10^{-12}$ with less than or about 200 times iterations, implying that the NCG method can quickly gain the dark solitons with very high accuracy.

When the numerical calculation is performing, the values of $a_1, a_2$ and $a_3$ for initial guesses are taken as $a_1=1.8$ for Fig. 3a, $a_1=0.45$ for Fig. 3c, $a_2=-a_3=-0.25$ for Fig. 3d and $a_2=-a_3=-0.45$ for Fig. 3f. The solitons given in Fig. 3 can be used as the initial guesses for NCG method to seek the dark solitons for other parameters. It is also can be seen easily from Fig. 3 that the amplitude of dark solitons decrease when three-body interaction strength $\left| {{g_2}} \right|$ increases.

**Figure 4:**
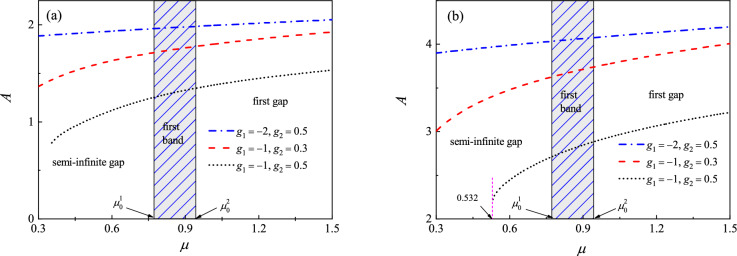
Amplitude of (**a**) kinks and (**b**) bubbles versus the chemical potential $\mu$ under different nonlinear interaction strength $g_1$ and $g_2$. The parameters are taken as $V_0=2, q=0.1$. The edge of first Bloch band is $\mu _0^1\approx 0.7726$ and $\mu _0^2\approx 0.9432$, respectively.

Figure 4a and b exhibit the amplitude of the kinks and bubbles versus the chemical potential $\mu$ under different nonlinear strength $g_1<0$ and $g_2>0$. From Fig. 4, we see that the dark solitons can be excited not only in the gap, but also in the band. This is quite different to the bright soliton discussed previously. The bright solitons exist in the gaps. On the other hand, in the semi-infinite gap, the dark solitons can be exited only for $\mu$ is greater than a certain value. Take an example, when $g_1=-1,\, g_2=0.5$, the kinks exist for $\mu >0.355$ and the bubbles for $\mu >0.532$, while the bright solitons exist for the entire gap. When $\mu$ moves toward the band edge, the amplitude of the bright soliton becomes smaller and smaller. However, this is not the fact for the dark solitons. From Fig. 4a and b, we see that the amplitude of the dark solitons increases monotonously as $\mu$ increases. It is known from “Bright solitons” section that the amplitude of the bright soliton decreases when the two-body interaction strength $|g_1|$ increases (other parameters are fixed). To our surprise, the amplitude of the dark solitons increases as $|g_1|$ increases.

**Figure 5:**
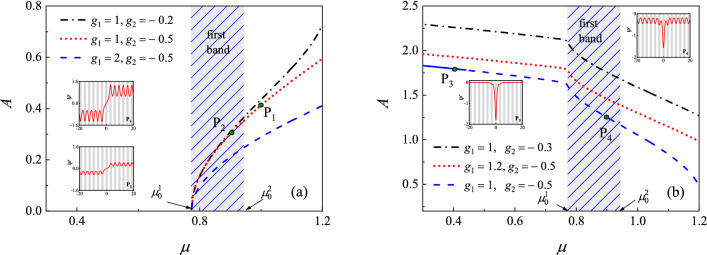
Amplitude of (**a**) kinks and (**b**) bubbles versus the chemical potential $\mu$ under different nonlinear interaction strength $g_1$ and $g_2$. The profiles of solitons at the marked points are shown in the subplots, where the shaded regions represent lattice sites, i.e., regions of low potential values *V*(*x*). The dashed line denotes the unstable solitons while the solid line denotes stable ones. The parameters are taken as $V_0=2, q=0.1$. The edge of first Bloch band is $\mu _0^1\approx 0.7726$ and $\mu _0^2\approx 0.9432$, respectively.

Figure 5a and b display the amplitude of the kinks and bubbles versus the chemical potential $\mu$ under different nonlinear strength $g_1>0$ and $g_2<0$. Here, we start computing from the points ${\mathrm {P}}_1$ (corresponding to $\mu =1$). The parameters for initial guess are $a_1=0.45, a_2=-a_3=-0.25$. The corresponding wave profiles at the marked points ${\mathrm {P}}_1-{\mathrm {P}}_4$ are shown in the subplots. The dashed lines denote the unstable

solitons while the solid line denotes stable ones (The stable property will be discussed later.). Again, we see that the dark solitons can be excited in the band. It can be seen from Fig. 5a that the kink solitons exist in the first band and first gap, but it can not be excited in the semi-infinite gap. The amplitude of the kink increases as chemical potential $\mu$ increases. When $\mu$ moves from ${\mathrm {P}}_1$ to ${\mathrm {P}}_2$, the amplitude becomes smaller distinctly. Both the amplitude of kink (modulation wave) and the amplitude of the Bloch wave (carrier wave) tend to zero as $\mu$ moves toward the lower edge of the first band. The kink soliton and the Bloch wave no longer exist when $\mu$ falls into the semi-infinite gap. From Fig. 5a, we suspect that the kink soliton may be regarded as a kink function, such as the tanh function, multiply by a Bloch wave function when the chemical potential $\mu$ near the lower edge of the first band.

When it comes to the bubbles (see Fig. 5b), the numerical results are quite different to the kink ones. One can see that the amplitude of the bubbles decreases as $\mu$ increases. When $\mu$ falls into the semi-infinite gap, the gray soliton reduces to an on-site-like bright soliton (see the wave profile at point ${\mathrm {P}}_3$). This is because the linearly periodic Bloch wave is prohibited in the semi-infinite gap. The profiles of this kind of solitons are similar to the bright solitons discussed before. However, they have different dynamical behaviors. The numerical results indicate that the on-site-like solitons are linearly unstable and dynamically unstable if $\mu$ is near the band edge. There is a critical value for $\mu$. The on-site-like solitons are linearly and dynamically stable only when $\mu$ is less than this critical value. For the parameters used in Fig. 5b ($g_1=1, g_2=-0.5$), the critical value for $\mu$ is about 0.438. This critical value depends on the nonlinear strength $g_1$ and $g_2$. From Fig. 5b, we suspect that the bubble soliton may be regarded as a superposition of a completely localized function and a Bloch wave function when $\mu$ near the lower edge of the first band.

### Multi-scale method

We now make a theoretical analysis on the solitons obtained in the above section. Considering that $\mu$ falls into the band-gap from the band edge $k=k_0$, $\mu _0=\mu (k_0)$, then $\psi (x)$ and $\mu$ can be expanded with multi-scale $X_0=x$, $X_1=\varepsilon ^{1/2}x$, that is

7 $$\begin{aligned} \psi (x)= & {} \psi _0(X_0,X_1)+{\varepsilon ^{{1 / 2}}}\psi _1(X_0,X_1)+\varepsilon \psi _2(X_0,X_1)+\cdots \end{aligned}$$

8 $$\begin{aligned} \mu= & {} \mu _0+\mu _2\varepsilon +\cdots \end{aligned}$$

where $\varepsilon =k-k_0$ is a small quantity. Suppose that $g_1=O(\varepsilon ), g_2=O(\varepsilon )$. Substituting the above expansions into Eq. (4), one can get

9 $$\begin{aligned} \varepsilon ^0: L_0\psi _0= & {} 0, \end{aligned}$$

10 $$\begin{aligned} {\varepsilon ^{{1 / 2}}}: L_0\psi _1= & {} \frac{\partial ^2\psi _0}{\partial X_0\partial X_1}, \end{aligned}$$

11 $$\begin{aligned} \varepsilon ^1: L_0\psi _2= & {} \frac{\partial ^2\psi _1}{\partial X_0\partial X_1}+\frac{1}{2}\frac{\partial ^2\psi _0}{\partial X_1^2}+\mu _2\psi _0-g_1\psi _0^3-g_2\psi _0^5, \end{aligned}$$

where $$L_0=-\frac{1}{2}\frac{\partial ^2}{\partial X_0^2}+V(X_0)-\mu _0$$.

Equation (9) is similar as Eq. (5), thus it possesses solution $\psi _0=B(X_1)p(X_0)$, where $p(X_0)$ is the carrier wave and $B(X_1)$ is the modulating wave. From Eq. (10), we then have $\psi _1=\frac{dB}{dX_1}H(X_0)$, where $H(X_0)$ is a periodic function, which satisfies $L_0H=\frac{dp}{dX_0}$. It’s easy to verify that the Fredholm condition is satisfied automatically. Substituting $\psi _0$ and $\psi _1$ into Eq. (11) yields

12 $$\begin{aligned} L_0\psi _2=\frac{d^2B}{dX_1^2}\left[ H'(X_0)+\frac{1}{2}p(X_0)\right] -g_1B^3p^3(X_0)-g_2B^5p^5(X_0)+\mu _2Bp(X_0). \end{aligned}$$

Applying the Fredholm condition to Eq. (12) leads to the following stationary nonlinear Schrödinger equation for $B(X_1)$^49^

13 $$\begin{aligned} -D\frac{\partial ^2B}{\partial X_1^2}-\mu _2B+g_1\alpha _1B^3+g_2\alpha _2B^5=0, \end{aligned}$$

where $D=\frac{1}{2}\frac{d^2\mu }{dk^2}\big |_{\mu =\mu _0}$, $\alpha _1= \frac{\int _0^{4K(q)}p^4(x)dx}{\int _0^{4K(q)}p^2(x)dx}$, $\alpha _2=\frac{\int _0^{4K(q)}p^6(x)dx}{\int _0^{4K(q)}p^2(x)dx}$. When the three-body interaction can be neglected, i.e. $g_2=0$, then Eq. (13) reduces to the stationary cubic NLSE. Note that $\alpha _1$ and $\alpha _2$ are always positive.

Equation (13) has a localized solitary wave solution, which reads

14 $$\begin{aligned} B(X_1)=\frac{\pm {\mathrm {sech}}(\beta _1 X_1)}{\sqrt{\beta _2+\beta _3{\mathrm {sech}^2}(\beta _1 X_1)}}, \end{aligned}$$

where $\beta _1=\sqrt{-2\mu _2/D}$, ${\beta _2} = \frac{{\sqrt{3g_1^2\alpha _1^2 + 16{g_2}{\alpha _2}{\mu _2}} }}{{2\sqrt{3} \left| {{\mu _2}} \right| }}$, ${\beta _3} = \frac{{{g_1}{\alpha _1} - 2{\beta _2}{\mu _2}}}{{4{\mu _2}}}$. Solution (14) is just the so-called “bright soliton”. If the three-body interaction is negligible, i.e. $g_2=0$, this soliton reduces to the bell-like soliton of the cubic NLSE.

**Figure 6:**
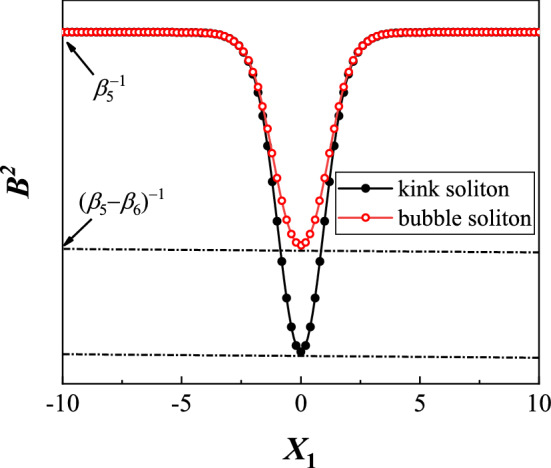
Sketch for the profiles of kink and bubble solitons.

Equation (13) also possesses the kink (the hole center has zero intensity) and bubble (having a nonzero value at the hole center) solitons^39,50,51^, which can be expressed as

15 $$\begin{aligned} B_{\mathrm {kink}}(X_1)=\frac{\pm {\mathrm {sinh}}(\beta _4 X_1)}{\sqrt{\beta _5{{\mathrm {sinh}}^2}(\beta _4 X_1)+\beta _6}}, \end{aligned}$$

and

16 $$\begin{aligned} B_{\mathrm {bubble}}(X_1)=\frac{\pm {\mathrm {cosh}}(\beta _4 X_1)}{\sqrt{\beta _5{{\mathrm {cosh}}^2}(\beta _4 X_1)-\beta _6}}, \end{aligned}$$

where $\beta _4=\sqrt{\frac{1}{D}\left( \mu _2-\frac{g_1\alpha _1}{2\beta _5}\right) }$, $\beta _5=\frac{g_1\alpha _1\pm \sqrt{g_1^2\alpha _1^2+4g_2\alpha _2\mu _2}}{2\mu _2}$ and $\beta _6= \frac{3g_1\alpha _1\beta _5^2+6g_2\alpha _2\beta _5}{3g_1\alpha _1\beta _5+4g_2\alpha _2}$. Figure 6 is a sketch for the profiles of kink and bubble, where $B^2$ is adopted rather than $B(X_1)$ itself. $B^2$ tends to a nonzero constant $1/\beta _5$ when $X_1\rightarrow \pm \infty$. At the center of the soliton, i.e. $X_1=0$, $B_{\mathrm {kink}}$ is zero but $B_{\mathrm {bubble}}$ is nonzero. It is also worth remarkable that the kink $B_{\mathrm {dark}}$ has a kink shape and the bubble $B_{\mathrm {bubble}}$ has a bell-like one. $\sqrt{\beta _5^{-1}}$ and $\left| \sqrt{\beta _5^{-1}}-\sqrt{(\beta _5-\beta _6)^{-1}}\right|$ can be regarded as the amplitude of the kink and bubble, respectively. When $\beta _6$ tends to zero, the bubbles can no longer be excited. For the kink, to avoid the singularity, it is required that both $\beta _5$ and $\beta _6$ should be positive. However, for the bubble, these conditions become $\beta _5>0$ and $\beta _6<\beta _5$, implying that $\beta _6$ can be negative. Note that $\beta _6$ equals to $\beta _5$ when $g_2=0$, suggesting that this kind of bubbles do not exist for the cubic NLSE. Therefore, the emergence of bubbles is just due to the effect of three-body interaction.

### Stability analysis

#### Linear stability analysis on bright solitons

We now numerically investigate the linear stability properties of solitons given by the NCG method. The perturbation carrier wave can be written as a Bogoliubov expansion

17 $$\begin{aligned} \Psi (x,t)=\{\psi (x)+[\upsilon (x)+w(x)]e^{\lambda t}+[\upsilon ^*(x)-w^*(x)]e^{\lambda ^*t}\}e^{-i\mu t}, \end{aligned}$$

where $\lambda$ is the eigenvalue of the normal mode, and $``*''$ denotes complex conjugation, $|\upsilon |,|w|\ll 1$ are infinitesimal normal-mode perturbations. Substituting this perturbed solution into Eq. (2) and linearizing, we find that these normal modes satisfy the following linear eigenvalue problem

18 $$\begin{aligned} \begin{bmatrix} 0 &{} L_1 \\ L_2 &{} 0 \end{bmatrix} \begin{bmatrix} \upsilon \\ w \end{bmatrix} =-i\lambda \begin{bmatrix} \upsilon \\ w \end{bmatrix} \end{aligned}$$

with $L_1=\frac{1}{2}\partial _{xx}+\mu -V(x)-g_1\psi ^2-g_2\psi ^4, L_2=\frac{1}{2}\partial _{xx}+\mu -V(x)-3g_1\psi ^2-5g_2\psi ^4$. General speaking, it is rather difficult to solve this linear eigenvalue problem analytically and exactly. However, we can solve it numerically and efficiently by the finite difference method or the Fourier collocation method^49^.

**Figure 7:**
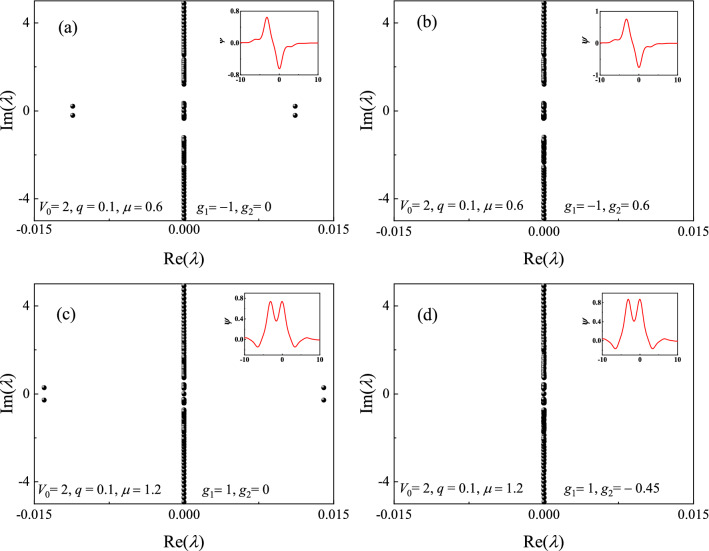
Stability spectra of gap solitons (**a**,**b**) in the semi-infinite gap and (**c**,**d**) in the first gap. The insets are the corresponding wave functions.

The numerical results indicate that all the on-site gap solitons of Eq. (4) are linearly stable. To indicate that the three-body interaction strength $g_2$ can indeed affect the stability of the off-site solitons, Fig. 7 shows the linear spectrum of off-site gap solitons under various of parameters. The gap soliton shown in Fig. 7a is linearly unstable ($g_1=-1, g_2=0$) while it is linearly stable in Fig. 7b ($g_1=-1, g_2=0.6$). Similarly, with different $g_2$, the soliton in Fig. 7c ($g_1=1, g_2=0$) is linearly unstable while it is linearly stable in Fig. 7d ($g_1=1, g_2=-0.45$) .

**Figure 8:**
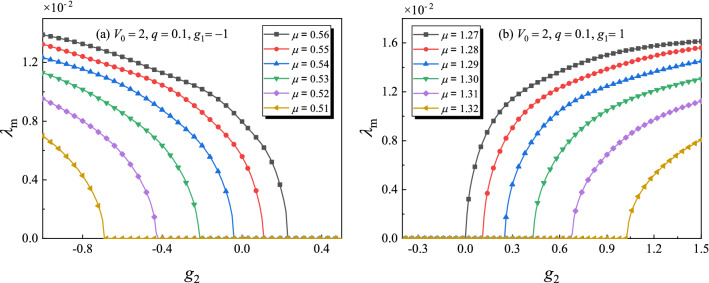
Maximum growth rate of perturbation $\lambda _m$ for the gap solitons versus the three-body interaction strength $g_2$ under different chemical potential $\mu$. The profiles of gap solitons are similar as Fig. 7b or d. (**a**) In the semi-infinite gap. (**b**) In the first gap.

To deeply understand the effects of $g_2$ on the stability properties, Fig. 8 shows the maximum growth rate of perturbation $\lambda _m=\max [{\mathrm{Re}}(\lambda )]$ for the gap solitons versus $g_2$ under different chemical potential $\mu$ ($V_0=2, q=0.1$). The corresponding profiles of gap solitons are similar as Fig. 7b or d. From Fig. 8, one can see that the unstable gap solitons become stable if $g_2$ increases over a critical value when other parameters are fixed. Thus, one can change the stability property of gap solitons by adjusting the three-body interaction strength in experiments.

#### Stability of dark solitons

**Figure 9:**
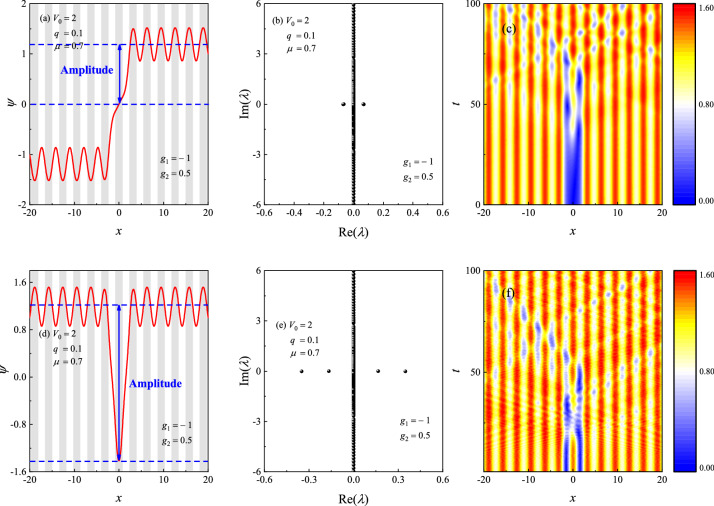
Profiles of (**a**) kink and (**d**) bubble in the semi-infinite gap. The shaded regions represent lattice sites, i.e., regions of low potential values *V*(*x*). (**b**,**e**) The linear stability spectrum of the dark solitons shown in (**a**) and (**d**), respectively. (**c**,**f**) Contour plots of $\left| \Psi (x,t)\right|$ for the dark solitons. The parameters are taken as $V_0=2, q=0.1, \mu =0.7, g_1=-1, g_2=0.5$.

Now, we numerically investigate the stability of the dark solitons discussed before. Figure 9a and d illustrate the profiles of dark solitons (red lines) given by NCG method for $V_0=2, q=0.1, \mu =0.7, g_1=-1, g_2=0.5$, in which $\mu$ lies in the semi-infinite gap. Figure 9b and e are the linear stability spectrum for the dark solitons shown in (a) and (d), respectively. One can see that both the kink and bubble are linearly unstable. To verify this conclusion, we have made the long-time evolution for Eq. (2), where the initial condition is taken as the random perturbed soliton obtained by the NCG method. The time-splitting Fourier Spectral method^52,53^ is adopted to make the time evolution. This method has high accuracy and can guarantee that the number of particles is conserved. Figure 9c and f are the contour plots of $|\Psi (x,t)|$. From which, it is clearly that the two dark solitons are dynamically unstable, which agrees well with the linear stability analysis. A natural question is whether there exists stable dark solitons (kink or bubble) in the semi-infinite gap. To answer this question, we have made lots of numerical calculations for various values of $g_1<0$ and $g_2>0$. Unfortunately, we failed to find the stable dark solitons for this case. In Ref.^54^, the authors found that the static bubble solitons are always unstable, which is also in well agreement with our conclusion.

**Figure 10:**
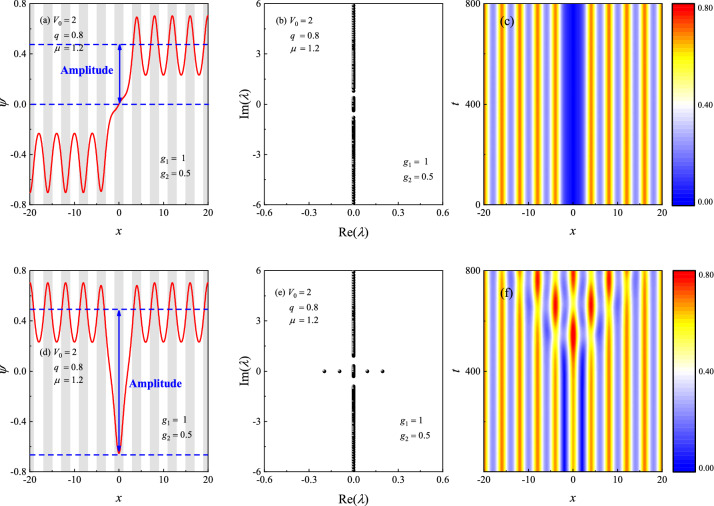
Profiles of (**a**) kink and (**d**) bubble in the first gap. The shaded regions represent lattice sites, i.e., regions of low potential values *V*(*x*). (**b**,**e**) The linear stability spectrum of the dark solitons shown in (**a**) and (**d**), respectively. (**c**,**f**) Contour plots of $\left| \Psi (x,t)\right|$ for the dark solitons. The parameters are taken as $V_0=2, q=0.8, \mu =1.2, g_1=1, g_2=0.5$.

Are there any stable dark solitons in the BEC system? Excitingly and interestingly, we indeed found stable kinks under certain parameters. Figure 10a and d illustrate the profiles of dark solitons (red lines) given by NCG method for $V_0=2, q=0.8, \mu =1.2, g_1=1, g_2=0.5$, in which $\mu$ lies in the first gap. Figure 10b and e are the linear stability spectrum for the dark solitons shown in (a) and (d), respectively. One can see that the kink soliton is linearly stable, while the bubble has two unstable modes, thus it is linearly unstable. Figure 10c and f are the contour plots of $|\Psi (x,t)|$. From which, it is clearly that the kink soliton is dynamically stable, while the bubble is dynamically unstable, which also agrees well with the linear stability analysis.

When the above nonlinear evolution is performed, the spacial interval is truncated from $(-\infty , +\infty )$ to (-32$T_x$, +32$T_x$), where $T_x$ is the periodicity of the external potential (${T_x} \approx \pi$ when $q=0.1$). Then the interval is divided into 8192 grids uniformly. We think that the length of the interval is large enough and the numerical method can give us the satisfactory results. On the other hand, we also think that the numerical error near the boundaries maybe large. So, we only pay attention on the interval $(-20, 20)$ when plotting the results (see Figs.  9,  10c and f). We believe that the numerical results have high accuracy in this smaller interval.

**Figure 11:**
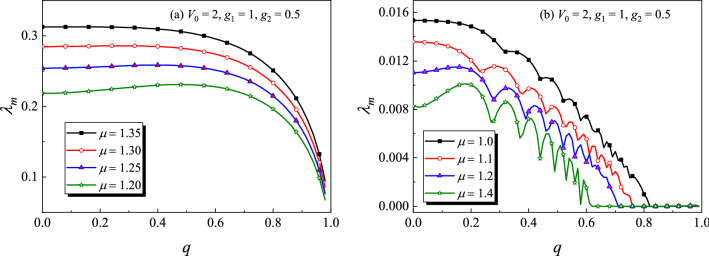
Maximum growth rate of perturbation $\lambda _m$ for dark solitons versus the modulus of external potential *q* under different chemical potential $\mu$. The profiles of dark solitons are similar as those shown in Fig. 9a or d. (**a**) bubble-like soliton (**b**) kink soliton.

From Figs. 9 and 10, we have the reason to suppose that the modulus *q* of the external potential may be an important parameter for the stability property of the dark solitons. To confirm this conclusion, Fig. 11 shows $\lambda _m$ for the dark solitons versus the modulus *q* under different chemical potential $\mu$. The corresponding profiles of dark solitons are similar as those shown in Fig. 9a or d. From Fig. 11a, we see that all the bubbles are linearly unstable. When *q* is near 1 (but less than 1), $\lambda _m$ decreases rapidly as *q* increases, implying that increasing the modulus of the external potential can weaken the instability of the bubble solitons. In Ref.^54^, the authors also found that the static bubble solitons are always unstable. Here, we have the same conclusion. However, for the kink solitons (see Fig. 11b), there exists a critical value of $q_\mu ^c$ for a given $\mu$. When $q<q_\mu ^c$, the kinks are linearly unstable, otherwise they are linearly stable. Take a instance, $q_\mu ^c \approx 0.7$ for $\mu =1.2$ and $q_\mu ^c\approx 0.61$ for $\mu =1.4$, suggesting that $q_\mu ^c$ depends upon $\mu$. Thus, one can change the stability properties of kinks by adjusting the modulus of external potential in experiments.

It is worth remarkable that $\lambda _m$ oscillates as *q* increases in Fig. 11b, which makes the figure does not look as “pretty good” as Fig. 11a. This is because, for kink solitons, $\lambda _m$ are relatively smaller ($\lesssim 10^{-3}$) than those of bubble-like solitons ($\sim 10^{-1}$), implying that the perturbation increases rather slowly. The oscillation may be due to the numerical errors. In fact, it is difficult to identify that a very small $\lambda _m$ is caused by the soliton instability or induced by the numerical errors. This trouble cannot be overcome even if one uses the long-time nonlinear evolution. In general, the soliton can be regarded as linearly stable within a relatively short period of time if $\lambda _m$ is small enough.

## Conclusion

In summary, we have analytically and numerically investigated the stability properties of bright and dark solitons in a quasi-1D BEC with three-body interaction loaded in a Jacobian elliptic sine potential. Bright and dark solitons are numerically found by the NCG method. A stationary nonlinear Schrödinger equation is derived to describe the profiles of solitons via the multi-scale technique. Linear stability analysis indicates that the three-body interaction strength has distinct effect on the stability properties. Especially, such a nonlinear system supports the so-called dark solitons (kink or bubble), which can be excited not only in the gap, but also in the band. The bubbles cannot be excited if the three-body interaction is absent and they are always unstable. Both stable and unstable kinks, depending on the physical parameters, can be excited in the BEC system.
